# Critical Analysis of Fixed-Dose Antibiotic Combinations Sold in Kinshasa—Democratic Republic of the Congo

**DOI:** 10.3390/antibiotics15030289

**Published:** 2026-03-12

**Authors:** Jocelyn Kakumba Mankulu, Dadit Kitenge Ive, Freddy Mugisho Kasago, Exaucé Mpuya Mpuya, Bertin K. Mfuamba, Jean-Pierre Mufusama Koy Sita, Patient Ciza Hamuli, Trésor Kimbeni Malongo, Jérémie Mbinze Kindenge, Jean-Marie Liesse Iyamba, Didi Mana Kialengila

**Affiliations:** 1 Laboratory of Drug Analysis, Faculty of Pharmaceutical Sciences, University of Kinshasa, Kinshasa P.O. Box 212, Democratic Republic of the Congo; 2 Laboratory of Pharmaceutical Organic and Analytical Chemistry, Faculty of Pharmaceutical Sciences, University of Kinshasa, Kinshasa P.O. Box 212, Democratic Republic of the Congo; 3 University Reference Center of Antimicrobial Resistance Surveillance (URC-AMRS), Faculty of Pharmaceutical Sciences, University of Kinshasa, Kinshasa P.O. Box 212, Democratic Republic of the Congo

**Keywords:** fixed-dose antibiotic combination, combi-packs, unsuitable antibiotics, low- and middle-income countries, pharmacies open to the public, Kinshasa-DR Congo

## Abstract

**Background**: Fixed-dose combination drugs (FDCs) are combinations of two or more active ingredients in a single dosage form. These formulations have proven effective in combating the development of resistance in diseases such as tuberculosis and malaria. Despite the benefits observed in the aforementioned cases, fixed-dose antibiotics combinations (FDACs) are increasingly raising questions about their rationality. This is the case for several FDACs listed in the AWaRe classification as not recommended, which unfortunately remain available on the pharmaceutical market, particularly in low- and middle-income countries like the Democratic Republic of Congo (DRC). **Objectives**: To identify the essential medicines available in pharmacies open to the public in the city of Kinshasa and to assess their inclusion in the DRC’s National List of Essential Medicines (NLEM) and in the World Health Organization’s (WHO) List of Essential Medicines (LEM). The rationality of the FDACs circulating in the city of Kinshasa were also evaluated based on the 2023 AWaRe classification. **Methods**: A cross-sectional and descriptive study was conducted between February and October 2025 in Kinshasa. For this purpose, fifty registered pharmacies open to the public were selected by systematic random sampling as the research sample. Data collection consisted of completing a data collection form after we had provided the pharmacies’ owners with the necessary explanations regarding the importance of the study and guaranteed their anonymity. **Results**: The controlled FDACs encountered comprised 27 specialties across 15 different formulations. Out of 15 formulations, 12 (80%) were included on the WHO list of non-recommended antibiotics and were not included in the DRC’s NLEM nor in the WHO’s LEM. Some had been withdrawn from the market in their countries of manufacture. Of the 15 FDACs evaluated for their rationality and compliance, the injectable FDACs presented problems related to the relevance and completeness of information contained on their packaging. On their primary packaging, there was a significant difference in the expiration dates of the powder and sterile water for injection contained in the combination pack, ranging from 6 to 36 months. Furthermore, the secondary packaging lacked data related to the sterile water for injection contained in the combination pack. In addition, several medications contained the same therapeutic combination. For injectable FDAC, for example, the combination Ceftriaxone-Sulbactam was represented by eight medications. For oral FDACs, the combination Sulfamethoxazole-Trimethoprim was represented by seven medications. Globally, 100% of these drug combinations originated from India. **Conclusions**: Fifteen varieties of FDACs were available in Kinshasa, most of which (80%) were unsuitable. It is important that public health authorities address this situation and develop stricter guidelines for granting marketing authorizations, particularly for FDACs.

## 1. Introduction

The irrational use (IU) of antimicrobials is a worrying global phenomenon that threatens public health. It is more characteristic of low- and middle-income countries where several factors facilitate access to antimicrobials (inappropriate prescriptions, sale of antibiotics without a prescription, non-adherence of patients to prescribed treatments, failure of healthcare workers to implement national guidelines for the management of common diseases, etc.) [1,2,3,4]. The IU of antimicrobials has a direct negative impact on the patient, who is exposed to inappropriate treatment in particular, and on the population in general because it promotes the development of antimicrobial resistance (AMR).

Several studies conducted worldwide, and in the DRC in particular, have shown a significant increase in cases of AMR associated with commonly used antibiotics [5,6,7,8].

AMR is the ability of a microorganism to resist the effects of antimicrobials. It is a problem that affects all regions of the world, but especially sub-Saharan African countries. Indeed, projections from the World Health Organization (WHO) show that AMR will cause more than 4,150,000 deaths by 2050 if appropriate measures are not taken [9].

Several strategies have been proposed to reduce the IU of antimicrobials and, consequently, AMR. Among these strategies, we can mention the following, among others:

- The implementation of the AWaRe classification of antibiotics, which encourages appropriate antibiotic use;
- The strengthening of pharmaceutical regulations to ensure prescribers adhere to international guidelines for the management of infectious diseases;
- Encouraging the use of drug combinations in dual or triple therapy whose efficacy has been scientifically proven and which are recommended by the WHO.

Indeed, fixed-dose combination (FDC) can be defined as drugs containing two or more active pharmaceutical ingredients in a single formulation. They represent a strategy for bypassing much of the early stages of drug development and reducing lengthy development times [6,10]. The synergistic mechanisms of action exploited in FDCs are likely to improve treatment response compared to monotherapy. This is the case with rifampicin + isoniazid in the management of tuberculosis, artemether/lumefantrine in the management of malaria, and tenofovir + lamivudine + efavirenz in the management of HIV, where they yield very good results [7,11].

Increasingly, fixed-dose antibiotic combinations (FDACs) are being used and encouraged in Africa in general, and in the DRC in particular, to combat AMR. To achieve this goal, it is essential that the antimicrobials used in combination be judiciously chosen and pharmacodynamically synergistic [12]. In the case of infections caused by drug-resistant Gram-negative bacilli, for example, the recommended broad-spectrum FDAC is a combination of α-lactam and α-lactamase inhibitors [13,14].

Globally, India is the largest consumer of antimicrobials and one of the countries with the highest rates of AMR [15,16]. To combat this AMR, India has the world’s largest selection of FDACs on the market [8]. Unfortunately, the existence of FDACs whose efficacy has not been proven, and which may therefore contribute to AMR, has been noted. This is why the WHO has expanded the AWaRe classification to include a list of non-recommended FDACs, and the Indian government banned approximately twenty-six FDACs in September 2018 due to their ineffectiveness [17]. The existence of these FDACs is neither based on evidence nor recommended by international reference manuals. Consequently, the WHO does not recommend their use in clinical practice [18]. This situation cannot leave African countries in general and the DRC in particular, as India is one of their major suppliers of medicines. Thus, in this study, we set ourselves the following objectives, among others:

- Compile a list of all available FDAC circulating in Kinshasa;
- Evaluate the completeness of FDAC in the NEML using the 2023 WHO’s LEM and the 2023 WHO AWaRe classification;
- Discuss the rationale behind some FDACs found on the market.

Indeed, it has been observed that the majority of injectable FDACs circulating in Kinshasa were presented in combi-pack, that is, a single secondary package containing a powder with the active ingredients (the associated antibiotics) and a bottle of sterile water for injection used to dissolve this powder. However, a recent study conducted in Kinshasa on injectable artesunates, which are also presented in combi-packs, revealed a serious problem with the traceability of their sterile water for injection due to their secondary packaging not meeting compliance criteria [18,19,20]. Hence, our fourth objective is the following:

- To conduct organoleptic examinations by evaluating the relevance and completeness of information contained in the primary and secondary packaging.

Until proven otherwise and to the best of our knowledge, no study has ever been conducted in Kinshasa on the quality of the FDACs sold there. It is in this context that this study was initiated to evaluate the rationality of FDACs in general, in order to raise awareness among the authorities about the quality of the medications they authorize. Thus, we aim to contribute to the fight against AMR and to the protection of public health.

## 2. Results

### 2.1. Injectable FDAC

#### 2.1.1. Evaluation of the Availability of Injectable FDAC

In total, we found on the market seven different injectable FDACs of different compositions, represented by 21 specialties marketed in the city-province of Kinshasa. Ceftriaxone-Sulbactam represented by eight specialties (38%) and Amoxicillin-Ac. Clavulanic acid by three products (14%) were available in all 50 pharmacies (100%), while Ceftriaxone-Tazobactam represented by four products (19%) and Piperacillin-Tazobactam by three products (14.5%) were available in 40 pharmacies (80%); Amoxicillin-Sulbactam represented by one product (5%) were available in 25 pharmacies (30%), and Cefoperazone-Sulbactam and Cefotaxime-Sulbactam represented by one product (5%) were available in 10 pharmacies (20%). The results of this study on the availability of injectable FDACs are summarized in Table 1.

#### 2.1.2. Assessment of the Relevance and Completeness of Information Contained on Packaging of Injectable FDAC in Combi-Packs

Packaging is an envelope of various shapes in which medications are contained. It must be hermetically sealed to avoid contact between the medicinal substance and external factors that could cause deterioration or influence the preservation or stability of the medication (humidity, light, atmospheric conditions, etc.). At this level, we can differentiate between primary and secondary packaging.

##### Assessment of the Relevance of Information Contained on Primary Packaging of FDAC Injectables

Primary packaging is the container that comes into direct contact with the medication. Its purpose is to contain, preserve, and protect the final product, particularly against impurities. The examination of the information available on this packaging showed that 100% of the packages examined contained information on the batch number, manufacturing date, and expiration date. However, a significant difference in shelf life was noted between the components of the same combi-pack, with this difference ranging from 6 to 36 months. The results of the examination of the primary packaging of injectable FDACs are presented in Table 2.

##### Assessment of Completeness of Information Contained on the Secondary Packaging of Injectable FDAC

This packaging contains the secondary packaging, designed to group several items into a single sales unit. It facilitates the handling of small products since they are grouped together in one package. A combi-pack is used when the secondary packaging contains two or more different products; in this case, the essential information from the primary packaging must also be included on the secondary packaging. This is the case for the secondary packaging of injectable FDACs, which contain two different products. The results of the completeness review of the information on these secondary packages showed that 100% of them contained the batch number, manufacturing date, and expiration date information only for the medication in the combination pack, and none (0%) contained this information for the sterile water for injection. These results are presented in Table 3.

### 2.2. Oral FDCA

In total, we worked on 10 different oral AADF compositions, represented by 27 specialties that are currently marketed in Kinshasa. Sulfamethoxazole-Trimethoprim represented by seven specialties (26%), Metronidazole-Norfloxacin by four (15%) and Amoxicillin-Ac. Clavulanic acid by four (15%) were present in all 50 pharmacies (100%). Ofloxacin-Ornidazole and Cefixime-Ofloxacin, each represented by three specialties (11%), were present in 35 pharmacies (70%). Ciprofloxacin-Ornidazole by two (7.5%) was present in 30 pharmacies (60%), Ciprofloxacin-Tinidazole and Amoxicillin-Sulbactam each represented by one specialty (3.7%) were present in 25 pharmacies (50%), and Levofloxacin-Ornidazole and Azithromycin-Fluconazole-Secnidazole each represented by one specialty (3.7%) were present in 15 pharmacies (30%). A total of 85% of all these specialties come from India. The results of the review of the availability of oral FDACs are summarized in Table 4.

### 2.3. Registration Status in the DRC’s NLEM and the WHO’s LEM

The NLEM is an essential tool that ensures the availability of well-selected medicines to address major health problems and promote better prescribing practices. This document, made available by the Minister of Health and recommended by the WHO as being updated every two years, is a valuable resource for all stakeholders at various levels of the healthcare system and for healthcare providers within care facilities [11]. The following was noted: among the 15 FDCA encountered in the field, 12 (80%) were not registered in the DRC NLEM nor in the WHO’s LEM, belonged to the WATCH group, and were not recommended by the AWaRe classification. Among the combinations not recommended by the WHO, we can mention the following, among others: Ceftriaxon (W) + Sulbactam (A), Ceftriaxon (W) + Tazobactam (W), Ciprofloxacin (W) + Ornidazole (A), Ciprofloxacin (W) + Tinidazole (A), Levofloxacin (W) and Ornidazole (A), Metronidazole (A) + Norfloxacin (W), and Ofloxacin (W) + Ornidazole (A).

Only three (20%) were registered in the DRC LNME, belonged to the ACCESS group, and were recommended by the AWaRe classification. Among them, we can mention Piperacillin (W) + Tazobactam (W), Amoxicillin (A) + Clavulanic acid (A) and Sulfamethoxazole (A) + Trimethoprim (A). These results are summarized in Table 5.

## 3. Discussion

The objective of our study was to identify the FDACs present in pharmacies open to the public in the city-province of Kinshasa, to verify their inclusion status in the DRC’s NLEM in WHO’s LEM, and to assess their rationality to the AWaRe classification. To this end, we conducted a systematic random sampling of 50 pharmacies open to the public, collecting data using a data collection form.

Our study of injectable FDAC revealed that 21 different pharmaceutical products on the market represent only seven combinations of two antibiotics. For example, the combination of ceftriaxone-sulfamethoxazole is represented by eight (38%) different products and the combination of ceftriaxone-tazobactam by four (19%). A similar observation was made regarding oral FDACs, where 14 drug combinations were represented by 27 pharmaceutical products. The combination of sulfamethoxazole-trimethoprim is contained in seven products and that of amoxicillin-clavulanic acid in four. This situation can be explained by weak enforcement of pharmaceutical regulations. Indeed, the granting of marketing authorizations should be subject to stricter conditions to prevent the existence of multiple products for the same drug composition. One or two high-quality products for a given drug formula would be more than sufficient.

Furthermore, it was found that 100% of injectable FDACs and 85% of oral FDA products marketed in Kinshasa come from India. Indeed, several published articles have shown that India has the largest number of FDACs on the market [8,18,21]. According to B. Bertone et al., out of 119 FDACs, 80 are found in India [8]. This situation has resulted in India currently having significant cases of AMR with respect to several antibiotics. To address this situation, in 2018, the Indian government banned 26 FDACs. Unfortunately, these banned products are still partially available on the Indian market or have been replaced by comparable alternatives after minor adjustments [21,22].

Moreover, FDACs banned in India are exported to other African and Asian countries. This export does not pose legal problems if the importing country does not have pharmaceutical regulations rejecting these FDACs [22,23]. This is unfortunately the case in the DRC, where some products already banned in India (the manufacturing country) are legal in the DRC (the consuming country). Examples of FDACs banned in India include Ofloxacin-Ornidazole and Norfloxacin-Metronidazole [24]. Yet our study clearly showed that these FDACs continue to circulate freely in the DRC. This pronounced trend of consumption of potentially inappropriate FDACs has been noted in several poor countries, including Egypt (9.6%), India (7.5%), and Pakistan (4%) [8].

The authorities responsible for protecting public health should be stricter regarding the quality of medicines before granting marketing authorizations. Indeed, the fact that the product being offered to us is already banned in its country of origin and not recommended by the WHO should be sufficient grounds for the authorities to raise concerns before granting marketing authorizations.

Regarding the information contained on the packaging of injectable FDACs presented in combi-packs, two major problems were noted that could compromise the conformity of these packages. Indeed, 100% of the secondary packaging contained information on the active ingredients and not on the sterile water for injection included in the combi-pack. This raises a traceability issue, as the sterile water for injection can be moved from one package to another at any time without leaving any trace. Furthermore, discrepancies in the expiration dates of the components contained in the primary packaging of the active ingredients and the sterile water for injection were observed. These discrepancies ranged from 6 to 36 months. This situation is concerning because while the powder of the active ingredients has already expired, the sterile water for injections remains valid, which will lead to the downgrading of the combi-pack even though the sterile water for injection can still be used. Manufacturers should organize themselves to package products with very close expiration dates in the same combi-pack or even sell them separately. This situation was also found in Kinshasa/DRC during a similar previous study conducted on injectable artesunates [24,25].

Since 2021, the WHO has recognized the need to expand its database on the rational use of antibiotics. Specifically, the AWaRe classification has been supplemented with the addition of a fourth category of drugs: “not recommended” antibiotics (*n* = 103). Several reasons justify this judicious choice by the WHO, including the concern for protecting public health. Non-recommended antibiotics have several shortcomings that discourage their use.

Our study revealed the presence of 15 FDACs that are legal in the DRC. Of these 15 FDACs, only 3 (20%) are registered on the NLEM and in WHO’s LEM. Twelve FDACs (80%) are not recommended according to AWaRe classification. These 12 non-recommended FDACs represent 11.5% (12/103) of all FDACs circulating worldwide. This high proportion of non-recommended FDACs circulating in the city-province of Kinshasa should attract the attention of health authorities due to the increased risks these products pose to public health. Similar situations have been reported in the literature, particularly in low-income countries. Indeed, a similar study conducted in Nigeria demonstrated that a third of its FDACs were imported from India, and in the majority of cases, these FDACs were not listed on the Nigerian NLEM nor in WHO LEM and were found on the list of non-recommended antibiotics in the AWaRe classification [26].

Furthermore, synergistic combinations of antibiotics can unexpectedly lead to a faster evolution of resistance than individual antibiotics [21,27]. This deplorable situation was noted with great concern in this study, where it was found that 12 out of 15 FDACs (80%), were classified in the Watch group. We know that this group consists of broad-spectrum antibiotics that can easily lead to the selection of resistant microorganisms. Previous studies had already shown that in Kinshasa, DRC, the trend was toward a greater use of Watch-class antibiotics than Access-class antibiotics. This already poses a serious risk to the future of antimicrobials in our country. Unfortunately, the free circulation of these FDACs, 80% of which are Watch-class, contributes to the rapid development of AMR. Health authorities must intervene to limit the importation of these high-risk products and to train prescribers in order to achieve the WHO objective of encouraging increased use (60%) of antibiotics in the Access group, as these have a narrow spectrum and therefore do not readily promote AMR.

In relation to public health and safety concerns, our study demonstrated that several FDACs still legally circulating in Kinshasa, DRC, pose problems. For the most part, there is virtually no data comparing their toxicity to that of monotherapy [28]. Several publications have highlighted the problems caused by FDACs. For example, the Ofloxacin-Ornidazole combination causes adverse effects in terms of cutaneous manifestations [29], while Ceftriaxone-Sulbactam exhibits significant activity only against class A beta-lactamases and has no efficacy against beta-lactamases B, C, or D. The 2:1 dose ratio in this FDCA is not based on any pharmacological justification. Moreover, a study conducted in Tanzania demonstrated that antagonism in the mechanism of action between combined active ingredients is common, as is the case when a bacteriostatic inhibitor of protein synthesis is combined with a bactericidal beta-lactam (e.g., doxycycline and ceftriaxone) [26,30,31]. All these weaknesses could promote the development of AMR.

Despite its widespread use in China as a first-line antibiotic against infectious diseases, a literature review has shown that its clinical use poses problems of coagulopathy and hemorrhage. Therefore, for its widespread use, further studies are needed [31,32].

The limitations of this study are related, among other things, to the data collection technique we used. Indeed, while we endeavored to cover the major pharmacies open to the public in the city of Kinshasa, it is possible that we inadvertently missed some FDACs available on the Kinshasa market. This is due to the possibility of numerous pharmaceutical specialties being available, the sampling frame, the exclusion of informal pharmacies, and the fact that we did not conduct laboratory quality control of the studied FDACs, the small size of our sample, and the lack of sales volume estimates for the non-recommended FDAC. Furthermore, this study was conducted primarily in Kinshasa, which somewhat limits its generalizability to the entire DRC.

## 4. Materials and Methods

### 4.1. Study Setting

This study was conducted in the city-province of Kinshasa, the capital of the DRC. The ongoing war in the eastern part of the country has led to a large population movement towards Kinshasa, resulting in a remarkable increase in its current population. This places Kinshasa among the three most populated cities in Africa. Due to its large population and the weak enforcement of pharmaceutical regulations, several pharmaceutical companies have found in Kinshasa a city where they can easily distribute their products of poor quality, sometimes even banned in their countries of origin. This dramatic situation makes Kinshasa the most suitable setting in the DRC for a pilot study related to the rationality assessment of FDACs. In addition, the majority of the population of Kinshasa lives below the poverty line, which exposes them to inferior quality health care [21].

### 4.2. Study Design, Sample Size, and Data Collection

A cross-sectional and descriptive study was conducted between February and October 2025 in the city-province of Kinshasa by graduating students from the Faculty of Pharmaceutical Sciences at the University of Kinshasa, following appropriate training.

Based on a list obtained from the Kinshasa Provincial Council of the Order of Pharmacists, approximately 50 pharmacies open to the public, distributed across the four districts of Kinshasa and holding operating licenses granted by the competent authorities of the Ministry of Public Health, were selected through systematic random sampling. The aim was to obtain 13 pharmacies per district to ensure adequate representation from each commune within the district. A data collection form enabled them to gather the necessary information on the availability of fixed-dose antibiotic combinations, their compositions, their marketing authorizations, their countries of origin and their inclusion in the DRC 2020 NLEM, the WHO’s 2023 LEM and the AWaRe 2023 classification.

The researchers began by providing detailed explanations about the purpose of the study, the potential impact of the results on public health, and the context in which the study was initiated—namely, as part of their final-year project. The pharmacists, having completed university training, readily accepted them, given their similar academic backgrounds. Indeed, the main step was convincing the supervising pharmacist to grant them access to their pharmacy, which was also easily accomplished since these final-year students were completing their internships in these public pharmacies.

We based our study on injectable forms, because they are the most used by private health centers that receive many patients for the treatment of common illnesses, and on oral forms, due to their widespread use for self-medication, thus representing a significant public health risk [4].

Furthermore, we conducted a thorough examination of the primary and secondary packaging of each injectable FDAC product available on the Kinshasa market, individually verifying the following information: the batch number, the manufacturing date, and the expiration date. Since injectable FDAC is presented in a combi-pack, all this information was checked on the two vials contained within the pack.

An FDAC was considered appropriate if it had a valid marketing authorization, was registered on NLEM, the WHO LEM, and was recommended by the AWaRe classification. Its packaging was considered compliant if it contained all the necessary information on both the primary and secondary packaging.

### 4.3. Ethical Considerations

As the study did not require any particular interaction with human or animal subjects, nor the recording of human data, we did not need authorization from the national ethics committee. However, we provided a detailed explanation of the study’s purpose to the managers of the pharmacies open to the public that we visited so that we could access the FDACs present in their establishments.

### 4.4. Data Analysis

The data collected were processed into a Microsoft Excel 2020 spreadsheet for processing and analysis. The results were presented using descriptive statistics in the form of frequencies, percentages, and tables.

## 5. Conclusions

A wide variety of available FDCAs were not registered in the 2020 DRC’s NLEM nor in the 2023 WHO’s LEM, with 80% not recommended by the 2023 WHO AWaRe classification, with many potential consequences for public health.

The presence on the market of several injectable and oral FDCA products was noted but for a limited number of therapeutic combinations. This deplorable situation stems from the authorities’ weakness in enforcing health regulations.

These results clearly demonstrate the need for policymakers and regulatory authorities of the pharmaceutical sector to develop more detailed guidelines governing the granting of marketing authorizations. This should limit the number of specialties for the same therapeutic composition and prevent the granting of marketing authorizations to non-recommended products following the WHO guidelines.

## Figures and Tables

**Table 1:** List of fixed-dose injectable antibiotic combinations collected.

N°	Produits Code	Number of Specialty	Availability in the Pharmacies	Country of Origin	Composition
1	CEF01–CEF08	8 (38%)	100%	India	Ceftriaxone + Sulbactam + sterile water pour injection
2	CEFO 01	1 (5%)	20%	India	Cefoperazone + Sulbactam + sterile water for injection
3	CEFT 01	1 (5%)	20%	India	Cefotaxime + Sulbactam + sterile water for injection
4	AmSulb01	1 (5%)	30%	India	Amoxicillin + Sulbactam + sterile water for injection
5	CEFTZ01-CEFTZ04	4 (19%)	80%	India	Ceftriaxone + Tazobactam + sterile water for injection
6	PITAZ 01-PITAZ03	3 (14%)	80%	India	Piperacillin + Tazobactam + sterile water for injection
7	Amclav01-Amclav03	3 (14%)	10%	India	Amoxicillin + Ac.Clavulanic + sterile water for injection

**Table 2:** Results of primary packaging examination.

N°	Drug Name	Comparison of Expiration Dates Drug vs. Sterile Water						
		Batch Number	Date of Manufacture	Expiration Date	Batch Number	Date of Manufacture	Expiration Date	Difference in Expiration Time (Months): Drug vs. Water
1	CEF01	OK	May 2023	October 2025	OK	March 2023	February 2027	16
2	CEF02	OK	April 2024	September 2026	OK	March 2024	February 2028	17
3	CEF03	OK	April 2022	April 2025	OK	March 2022	February 2027	22
4	CEF04	OK	June 2024	June 2026	OK	July 2024	June 2029	36
5	CEF05	OK	November 2023	October 2025	OK	October 2023	September 2028	35
6	CEF06	OK	January 2024	December 2025	OK	May 2023	April 2028	30
7	CEF07	OK	January 2023	June 2025	OK	January 2023	January 2026	6
8	CEF08	OK	January 2023	June 2025	OK	May 2022	April 2026	10
9	CEFOP 01	OK	February 2023	July 2025	OK	June 2022	May 2026	10
10	CEFOT01	OK	April 2024	May 2026	OK	March 2024	February 2029	30
11	AmSulb01	OK	December 2024	October 2026	OK	August 2024	July 2029	33
12	CEFTZ01	OK	October 2023	March 2026	OK	August 2023	July 2027	34
13	CEFTZ02	OK	October 2023	September 2025	OK	August 2023	July 2028	22
14	CEFTZ03	OK	April 2024	September 2026	OK	April 2024	March 2028	18
15	CEFTZ04	OK	July 2025	January 2028	OK	March 2023	February 2029	12
16	PITAZ 01	OK	April 2024	March 2026	OK	April 2024	March 2029	36
17	PITAZ 02	OK	October 2023	September 2025	OK	August 2023	July 2027	22
18	PITAZ03	OK	August 2024	January 2027	OK	February 2024	January 2028	12
19	Amclav01	OK	August 2024	March 2026	OK	March 2023	February 2027	12
20	Amclav02	OK	July 2024	February 2026	OK	March 2023	February 2027	12
21	Amclav03	OK	October 2023	February 2025	OK	October 23	September 2027	30

**Table 3:** Results of the completeness of information on the secondary packaging.

N°	Products Code	Drug Data t		
		Batch Number	Date of Manufacture	Expiration Date
1	CEF01	OK	May 2023	October 2025
2	CEF02	OK	April 2024	09 2026
3	CEF03	OK	April 2022	April 2025
4	CEF04	OK	June 2024	June 2026
5	CEF05	OK	November 2023	October 2025
6	CEF06	OK	January 2024	December 2025
7	CEF07	OK	January 2023	June 2025
8	CEF08	OK	January 2023	June 2025
9	CEFP 01	OK	February 2023	July 2025
10	CEFT 01	OK	April 2024	May 2026
11	CEFTZ01	OK	October 2023	March 2026
12	CEFTZ02	OK	October 2023	September 2025
13	CEFTZ03	OK	April 2024	September 2026
14	CEFTZ04	OK	July 2025	January 2028
15	PITZ 01	OK	April 2024	March 2026
16	PITZ 02	OK	October 2023	September 2025
17	PITAZ03	OK	August 2024	January 2027
18	Amclav01	OK	August 2024	March 2026
19	Amclav02	OK	July 2024	February 2026
20	Amclav03	OK	October 2023	February 2025
21	Amclav04	OK	December 2024	October 2026

**Table 4:** Availability assessment of oral FDACs.

N°	Products Codes	Number of Specialty	Availability in the Pharmacies	Country of Origin	Composition
1	CEF01-CEF03	3 (11%)	70%	India	Cefixime + Ofloxacin
2	LEV01	1 (3.7%)	30%	India	Levofloxacin + Ornidazole
3	TRIK01	1 (3.7%)	30%	India	Azithromycin + Fluconazole + Secnidazole
4	OFL 01-OFL 03	3 (11%)	70%	India	Ofloxacine + Ornidazole
5	MET 01-MET 04	4 (15%)	100%	India	Métronidazole + Norfloxacin
6	CIPR01-CIPR02	2 (7.5%)	60%	India	Ciprofloxacin, Ornidazole
7	CIPTR01	1 (3.7%)	50%	India	Ciprofloxacin, Tinidazole
8	AMX01	1 (3.7%)	50%	India	Amoxicillin, Sulbactam
9	MLC 01-MCL04	4 (15%)	100%	India	Amoxicillin et acide Clavulanic
10	SUL 01-SUL 03	3 (11%)	100%	India	Sulfamethoxazole, Trimethoprim
11	SUL 04	1 (3.7%)		Germany	
12	SUL 05	1 (3.7%)		France	
13	SUL 06	1 (3.7%)		Pakistan	
14	SUL 07	1 (3.7%)		China	

Note: Certain combinations of antibiotics with antifungals and/or antiprotozoals (e.g., azithromycin + fluconazole + secnidazole) are considered as antibiotic combinations according to the WHO AWaRe classification [17].

**Table 5:** Registration status of FDCA in the DRC’s NLEM and the WHO’s LEM.

**N°**	**NOT LISTED ON THE WHO LEM**	**DRC’s NLEM**	**AWaRe Group**	**AWaRe** **Classification**
1	Azithromycin (W), Fluconazole et Secnidazole (A)	Not included 80% (12/15)	WATCH (W) 80% (12/15)	Not recommended 80% (12/15)
2	Cefixime (W) et Ofloxacin (W)			
3	Cefoperazone (W) + Sulbactam (A)			
4	Cefotaxime (W) + Sulbactam (A)			
5	Ceftriaxon (W) + Sulbactam (A)			
6	Ceftriaxon (W) + Tazobactam (W)			
7	Ciprofloxacin (W) + Ornidazole (A)			
8	Ciprofloxacin (W) + Tinidazole (A)			
9	Levofloxacin (W) et Ornidazole (A)			
10	Métronidazole (A) + Norfloxacin (W)			
11	Ofloxacin (W) + Ornidazole (A)			
12	Amoxicillin (A) + Sulbactam (A)		ACCESS (A) 20% (3/15)	
	**LISTED IN WHO INSCRIT SUR LA LME DE L’OMS**			
13	Piperacillin (W) + Tazobactam (W)	Included 20% (3/15)	WATCH (W)	Recommended 20% (3/15)
14	Amoxicillin (A) + Clavulanic acid (A)		ACCESS (A)	
15	Sulfamethoxazole (A) + Trimethoprim (A)			

## Data Availability

All data related to this research are contained within this paper.
